# CD1d-Restricted Type II NKT Cells Reactive With Endogenous Hydrophobic Peptides

**DOI:** 10.3389/fimmu.2018.00548

**Published:** 2018-03-15

**Authors:** Yusuke Nishioka, Sakiko Masuda, Utano Tomaru, Akihiro Ishizu

**Affiliations:** ^1^Graduate School of Health Sciences, Hokkaido University, Sapporo, Japan; ^2^Faculty of Health Sciences, Hokkaido University, Sapporo, Japan; ^3^Department of Pathology, Faculty of Medicine, Graduate School of Medicine, Hokkaido University, Sapporo, Japan

**Keywords:** CD1d, NKT cell, hydrophobic peptide, sulfatide, glycolipid

## Abstract

NKT cells belong to a distinct subset of T cells that recognize hydrophobic antigens presented by major histocompatibility complex class I-like molecules, such as CD1d. Because NKT cells stimulated by antigens can activate or suppress other immunocompetent cells through an immediate production of a large amount of cytokines, they are regarded as immunological modulators. CD1d-restricted NKT cells are classified into two subsets, namely, type I and type II. CD1d-restricted type I NKT cells express invariant T cell receptors (TCRs) and react with lipid antigens, including the marine sponge-derived glycolipid α-galactosylceramide. On the contrary, CD1d-restricted type II NKT cells recognize a wide variety of antigens, including glycolipids, phospholipids, and hydrophobic peptides, by their diverse TCRs. In this review, we focus particularly on CD1d-restricted type II NKT cells that recognize endogenous hydrophobic peptides presented by CD1d. Previous studies have demonstrated that CD1d-restricted type I NKT cells usually act as pro-inflammatory cells but sometimes behave as anti-inflammatory cells. It has been also demonstrated that CD1d-restricted type II NKT cells play opposite roles to CD1d-restricted type I NKT cells; thus, they function as anti-inflammatory or pro-inflammatory cells depending on the situation. In line with this, CD1d-restricted type II NKT cells that recognize type II collagen peptide have been demonstrated to act as anti-inflammatory cells in diverse inflammation-induction models in mice, whereas pro-inflammatory CD1d-restricted type II NKT cells reactive with sterol carrier protein 2 peptide have been demonstrated to be involved in the development of small vessel vasculitis in rats.

## Introduction

NKT cells are first reported as T cells that share surface markers of NK cells and recognize antigens presented by the major histocompatibility complex (MHC) class I-like molecule CD1d (1). Because subsequent studies have demonstrated that there are CD1d-restricted T cells that do not express NK markers and that conventional T cells express NK markers when activated, CD1d restriction rather than the expression of NK markers is considered to be a critical feature of NKT cells (2, 3). Currently, NKT cells are defined as T cells that recognize antigens presented by MHC class I-like molecules, including CD1d.

CD1d is a CD1 family member that can present hydrophobic antigens (e.g., glycolipids) (4). Whereas the CD1 family includes CD1a, CD1b, CD1c, CD1d, and CD1e in humans, rodents express only CD1d but not CD1a, CD1b, CD1c, and CD1e (5, 6).

CD1d-restricted NKT cells stimulated by antigens can activate or suppress other immunocompetent cells through an immediate production of a large amount of cytokines. Thus, these cells are regarded as immunological modulators. They are classified into two subsets, namely, type I and type II, according to the different usage of T-cell receptors (TCRs) (7, 8).

CD1d-restricted type I NKT cells are also called invariant NKT cells because of their usage of the limited α-chain (Vα24-Jα18 in humans and Vα14-Jα18 in rodents) with no or very few nucleotide insertions in the complementarity-determining region 3 that constitutes TCR. These cells can recognize a glycolipid, α-galactosylceramide (α-GalCer) (9–11). Based on this evidence, α-GalCer-loaded CD1d tetramers are employed to detect CD1d-restricted type I NKT cells. Up to now, several microbial and endogenous lipids have been identified as antigens recognized by CD1d-restricted type I NKT cells (12).

On the contrary, CD1d-restricted type II NKT cells use diverse TCRs (13) and react with a wide variety of antigens, including microbial and endogenous glycolipids and phospholipids and endogenous hydrophobic peptides, but do not recognize α-GalCer. Due to the difficulty in preparation of ligand-loaded CD1d tetramers to detect CD1d-restricted type II NKT cells specifically, studies on these cells fall behind those on CD1d-restricted type I NKT cells. However, in terms of the abundance of CD1d-restricted type II NKT cells compared to CD1d-restricted type I NKT cells in humans, it is worthy of understanding their physiological and pathological roles (14, 15). In this review, we focus particularly on CD1d-restricted type II NKT cells that recognize endogenous hydrophobic peptides presented by CD1d.

## Antigens Recognized by CD1d-Restricted Type II NKT Cells

Previous studies have demonstrated that CD1d-restricted type II NKT cells recognize microbial and endogenous glycolipids and phospholipids. Tatituri et al. identified *Mycobacterium tuberculosis*-derived phosphatidylglycerol (PG), diphosphatidylglycerol (cardiolipin), and phosphatidylinositol as exogenous antigens recognized by CD1d-restricted type II NKT cells (16). More recently, Wolf et al. have demonstrated that Gram-positive *Listeria monocytogenes*-derived PG is also recognized by CD1d-restricted type II NKT cells (17). It has been demonstrated that CD1d-restricted type II NKT cells can recognize lysophosphatidylcholine species (18) and sulfatides, which are endogenous glycolipids abundant in the central nervous system (19). Nair et al. identified two types of CD1d-restricted type II NKT cells in patients with Gaucher’s disease (20). These cells recognize β-glucosylceramide and glucosylsphingosine. Rhost et al. have demonstrated that β-GalCer is also recognized by CD1d-restricted type II NKT cells (21).

Although CD1d mainly presents lipid antigens, such as glycolipids, earlier studies depicted that it can present hydrophobic peptides (22, 23). In 2011, Liu et al. reported that type II collagen peptide could be an autoantigen recognized by CD1d-restricted NKT cells (24). Although they did not declare the subtype of type II collagen-reactive CD1d-restricted NKT cells in the paper, it appears to be a type II phenotype in terms of the antigen property. More recently, our group demonstrated that the hydrophobic peptide derived from sterol carrier protein 2 (SCP2), an intracellular lipid transporter, could be presented by CD1d and then recognized by CD1d-restricted type II NKT cells (25). Girardi et al. conducted X-ray analyzes of binding formation of CD1d and peptide antigens (26). The results demonstrated that the binding mode of peptides and CD1d is obviously different from that of glycolipids and CD1d but rather resemble that of peptides and MHC.

Castano et al. reported that peptide antigens that contain a motif of [F/W]XX[I/L/M]XXW can bind to CD1d (22). However, neither type II collagen peptide PPGANGNPGPAGPPG (24) nor SCP2 peptide FFQGPLKITGNMGLA (25) contains this motif. Furthermore, the membrane proximal external region of HIV-1 glycoprotein 41 peptide that contains this motif cannot bind to CD1d (26). The collective findings suggest that it is not easy to generalize the motif that represents the binding potential to CD1d. Not only amino acid sequences but also electric charge and peptide length could influence the binding to CD1d.

## Function of CD1d-Restricted Type I NKT Cells

It has been demonstrated that the development of experimental autoimmune encephalomyelitis (EAE) and uveitis is enhanced in CD1d-restricted type I NKT cell-deficient Jα18 knockout mice (27, 28). In addition, the activation of CD1d-restricted type I NKT cells protects non-obese diabetes (NOD) mice from developing insulitis (29). These findings suggest an anti-inflammatory aspect of CD1d-restricted type I NKT cells in these murine autoimmune diseases. On the contrary, Jahng et al. reported that the simultaneous activation of CD1d-restricted type I NKT cells and myelin-reactive T cells exacerbated the progression of EAE (30), and Griseri et al. reported that CD1d-restricted type I NKT cells accelerated insulitis in the CD8 T cell-mediated diabetes model (31). These controversial results might be attributed to the experimental conditions. Interestingly, it has been demonstrated that CD1d-restricted type I NKT cells protect from Th1-mediated inflammation (32–34) but exacerbate Th2-mediated inflammation (35–37).

The following studies suggest a rather pro-inflammatory than anti-inflammatory phenotype of CD1d-restricted type I NKT cells. Chiba et al. reported that CD1d-restricted type I NKT cell number was increased in arthritic joints in the collagen-induced arthritis model (33). In another antibody-induced arthritis model, CD1d-restricted type I NKT cells augmented inflammation in the joints by suppressing the production of the anti-inflammatory transforming growth factor β (TGF-β) (34). CD1d-restricted type I NKT cells accelerated inflammation also in cholangitis model (38). Furthermore, Kumar et al. reported that CD1d-restricted type I NKT cells acted as an inflammation promoter in liver injury by activating NK cells that kill hepatocytes through Fas/Fas ligand (FasL) interaction as well as by producing the pro-inflammatory cytokines (39–41). The more recent study demonstrated that the activation of CD1d-restricted type I NKT cells in the lungs by *Francisella tularensis* induced tularemia-like disease in mice (42).

In tumor immunity, CD1d-restricted type I NKT cells are also associated with the promotion of immune response against tumors (43). For instance, it has been demonstrated that the activation of CD1d-restricted type I NKT cells increased survival in mice bearing B16 melanoma (44, 45). Subsequent studies have revealed that a large amount of interferon-γ released from activated CD1d-restricted type I NKT cells is pivotal for tumor protection (46, 47).

## Function of CD1d-Restricted Type II NKT Cells

The function of CD1d-restricted type II NKT cells has been investigated mainly by the following methods: (1) *in vivo* and/or *in vitro* stimulation by sulfatides; (2) observation of the difference in phenotype between CD1d knockout mice, which lack whole CD1d-restricted NKT cells, and Jα18 knockout mice, which solely lack CD1d-restricted type I NKT cells; and (3) use of 24αβ transgenic mice that carry the CD1d-restricted type II NKT cell-derived TCR gene. The stimulation of CD1d-restricted type II NKT cells by sulfatides resulted in anti-inflammatory effects on liver injury (39, 40). Kwiecinski et al. demonstrated that sulfatide-stimulated CD1d-restricted type II NKT cells attenuated sepsis induced by *Staphylococcus aureus* (48). Concerning these mechanisms, some studies have demonstrated that sulfatide-stimulated CD1d-restricted type II NKT cells suppressed the activation of pro-inflammatory type I NKT cells (39, 49, 50).

Terabe et al. and Renukaradhya et al. independently conducted experiments employing CD1d knockout and Jα18 knockout mice, and they both demonstrated that CD1d-restricted type II NKT cells downregulated cancer immunosurveillance (51, 52). Furthermore, other experiments that employed CD1d knockout and Jα18 knockout mice revealed that CD1d-restricted type II NKT cells attenuated the development of graft-versus-host disease after bone marrow transplantation (53).

Cardell et al. generated the CD1d-restricted type II NKT cell hybridoma VIII24 from MHC class II knockout mice (54). Skold et al. developed 24αβ mice that carried the Vα3.2-Vβ9 gene derived from the TCR of VIII24 hybridoma (55). Duarte et al. transduced the Vα3.2-Vβ9 gene into NOD mice and established 24αβ/NOD mice (56). These mice exhibited a decrease in the incidence of diabetes compared to the parent NOD mice. Furthermore, Liao et al. generated 24αβ/CD1dTg mice that overexpressed CD1d, and demonstrated that these mice spontaneously developed colitis underlying dysregulated differentiation of CD1d-restricted Vα3.2-Vβ9^+^ type II NKT cells in the thymus (57).

**Figure 1:**
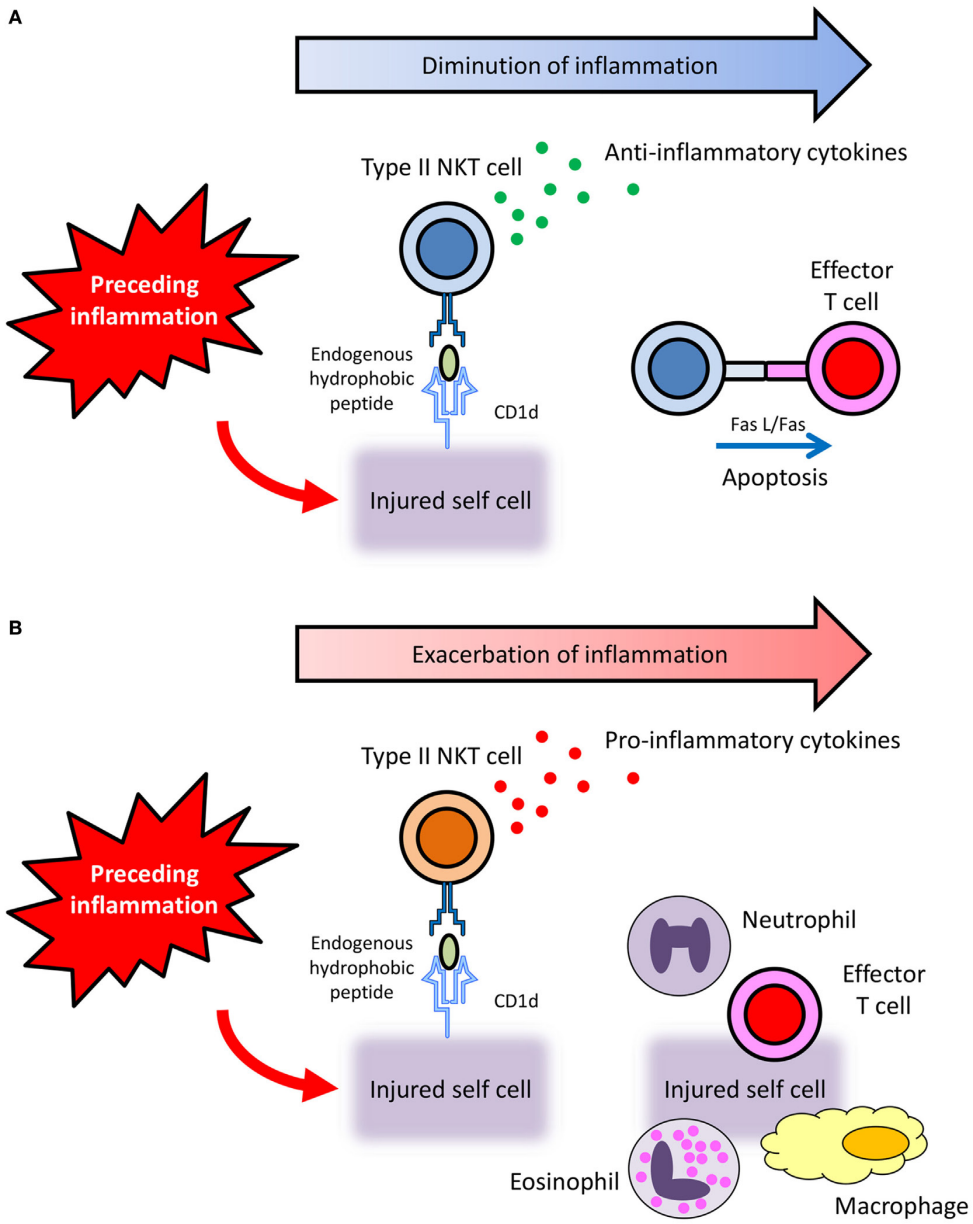
Hypothetical diverse roles of CD1d-restricted type II NKT cells that recognize endogenous hydrophobic peptides. **(A)** Preceding inflammation sometimes results in the injury of own tissues. Under such situation, injured cells then present hydrophobic autoantigens, probably peptides, on their CD1d to activate CD1d-restricted type II NKT cells. Thereafter, activated CD1d-restricted type II NKT cells function to diminish inflammation by producing anti-inflammatory cytokines and by inducing apoptosis of effector cells *via* Fas/FasL interaction. **(B)** When CD1d-restricted type II NKT cells activated by endogenous hydrophobic peptides produce pro-inflammatory cytokines, the inflammation is exacerbated.

The study published by Liu et al. (24) is noteworthy. They reported that type II collagen peptide-reactive CD1d-restricted NKT cells suppressed autoimmune arthritis by producing TGF-β, an anti-inflammatory cytokine, and by inducing apoptosis of effector cells through Fas/FasL interaction. This report encouraged us to make the following hypothesis: preceding inflammation sometimes results in the injury of own tissues. Under such situation, injured cells then present hydrophobic autoantigens, probably peptides, on their CD1d to activate CD1d-restricted type II NKT cells. Thereafter, activated CD1d-restricted type II NKT cells function to diminish inflammation by producing anti-inflammatory cytokines and by inducing apoptosis of effector cells *via* Fas/FasL interaction (Figure 1A).

## Involvement of CD1d-Restricted Type II NKT Cells in Immune-Related Inflammatory Diseases

It has been demonstrated that CD1d-restricted type II NKT cells play critical roles in the development of liver injury in an acute hepatitis B transgenic murine model (58). In addition, the activation of CD1d-restricted type II NKT cells is accompanied by conventional T cell activation and pro-inflammatory cytokine production, leading to an enhancement of hepatic injury in murine autoimmune hepatitis models (59).

In patients with ulcerative colitis (UC), sulfatide-reactive CD1d-restricted type II NKT cells in lamina propria mononuclear cells are increased compared to healthy controls and patients with Crohn’s disease (60). Moreover, sulfatide stimulation induced pathogenic interleukin (IL)-13 production and IL-13Rα2 expression on CD1d-restricted type II NKT cells from UC patients but not from healthy controls or patients with Crohn’s disease. These findings are consistent with the previous report indicating that CD1d-restricted type II NKT cells with dysregulated differentiation is pathogenic in the murine colitis model (57). A recent study using CD1d-deficient (CD1d knockout) and CD1d-restricted type I NKT cell-deficient (Jα18 knockout) mice has also demonstrated that pro-inflammatory type II NKT cells are involved in dextran sulfate sodium-induced colitis in mice (61).

More recently, our group has revealed the involvement of pro-inflammatory type II NKT cells that are reactive with the endogenous SCP2 peptide in the pathogenesis of small vessel vasculitis in rats (25, 62) (Figure 1B). CD1d-restricted type II NKT cells activated by the SCP2 peptide function to enhance inflammation by producing pro-inflammatory cytokines. Although further studies are needed to clarify the precise mechanism, the involvement of pro-inflammatory CD1d-restricted type II NKT cells that recognize endogenous hydrophobic peptide is worthy of attention in the pathogenesis of immune-related inflammatory diseases.

## Author Contributions

All authors listed have made a substantial, direct, and intellectual contribution to the work and approved it for publication.

## Conflict of Interest Statement

The authors declare that the research was conducted in the absence of any commercial or financial relationships that could be construed as a potential conflict of interest.
